# Supplementary material for: Multi-omics analyses reveal fecal microbial community and metabolic alterations in finishing cattle fed probiotics-fermented distiller’s grains diets
Source: Microbiol Spectr. 2025 Apr 11;13(5):e00721-24. doi: 10.1128/spectrum.00721-24 (PMC12054032; doi:10.1128/spectrum.00721-24)
Supplement: Supplemental Material — Supplemental tables and figures. [file spectrum.00721-24-s0001.docx]

Table S1 The composition and nutrient levels of concentrates in the experimental diets^1^ (%, on dry matter basis).

| **Items** | **CON** | **FDG-10%** | **FDG-20%** |
| --- | --- | --- | --- |
| **Ingredient（%）** |  |  |  |
| FDG | 0.00 | 10.00 | 20.00 |
| Corn | 35.75 | 29.25 | 22.75 |
| Wheat bran | 5.72 | 4.68 | 3.64 |
| Soybean meal | 11.00 | 9.00 | 7.00 |
| Calcium carbonate, 1% | 0.385 | 0.315 | 0.245 |
| Calcium hydrogen phosphate | 0.055 | 0.045 | 0.035 |
| Sodium chloride | 0.44 | 0.36 | 0.28 |
| Sodium bicarbonate | 0.825 | 0.675 | 0.525 |
| Premix^2^ | 0.825 | 0.675 | 0.525 |
| Total | 100.00 | 100.00 | 100.00 |
| **Nutrition levels^3^** |  |  |  |
| Metabolic energy, ME, MJ/Kg | 10.98 | 10.33 | 9.72 |
| Crude protein, CP /% | 15.40 | 18.10 | 20.13 |
| Neutral detergent fiber, NDF /% | 12.15 | 20.77 | 29.44 |
| Acid detergent fiber, ADF /% | 4.85 | 12.12 | 19.30 |
| Calcium, Ca /% | 0.40 | 0.51 | 0.48 |
| Phosphorus, P /% | 0.42 | 0.54 | 0.62 |

^1^ CON, FDG-10%, and FDG-20% represent the group without FDG supplementation, the group with 10% FDG substituting for 10% concentrate, and the group with 20% FDG substituting for 20% concentrate, respectively.

^2^ The premix provided the following amount of vitamins and minerals per kilogram diet: vitamin A, 100-500 KU; vitamin D3, 50-200 KIU; vitamin E, ≥ 500 IU; Fe (FeSO4), 1000-10000 mg; Cu (CuSO4), 500-1500 mg; Zn (ZnSO4), 1000-5000 mg; Mn (MnSO4), 1000-7000 mg; Se (Na2SeO3), 5-20 mg; Co (CoCl₂), 5-50 mg; I₂(KI), 20-100 mg.

^3^ Nutrient levels are measured values.

Table S2 Effects of dietary FDG on the growth performance of finishing cattle

| **Item** | **Groups** | | | **SEM** | ***P*-Value** |
| --- | --- | --- | --- | --- | --- |
|  | **CON** | **FDG-10%** | **FDG-20%** |  |  |
| ADG, Kg | 0.92±0.04^a^ | 0.96±0.05^a^ | 0.87±0.05^a^ | 0.04 | 0.75 |

ADG: average daily gain.

CON, FDG-10%, and FDG-20% represent the group without FDG supplementation, the group with 10% FDG substituting for 10% concentrate, and the group with 20% FDG substituting for 20% concentrate, respectively.

Data are presented as means ± SEM (n=6), and the same row with same superscript indicates no significant difference (*P* > 0.05).

Table S3 The significantly different metabolites in positive (pos) ion modes.

| **Metabolites** | **log2(FC)** | ***P*** | **VIP** |
| --- | --- | --- | --- |
| **FDG-10% Vs CON** |  |  |  |
| Sinapine | 20.652 | 0.000 | 20.280 |
| Silodosin | 7.379 | 0.000 | 14.082 |
| .gamma.-glu-cys | 20.165 | 0.000 | 13.734 |
| N-acetyl-d-galactosamine | 0.531 | 0.047 | 9.864 |
| D-glucosaminic acid | 2.077 | 0.004 | 9.630 |
| Dethiobiotin | 6.210 | 0.000 | 9.526 |
| Citalopram | 4.242 | 0.000 | 8.330 |
| 2-chloro-2',6'-diethylacetanilide | 0.508 | 0.001 | 8.168 |
| Celaxanthin | 0.714 | 0.002 | 7.215 |
| Lenalidomide | 3.832 | 0.001 | 7.064 |
| DL-tyrosine | 3.520 | 0.000 | 7.026 |
| 4,4'-diaminodiphenylmethane | 7.614 | 0.000 | 6.658 |
| Vasicinone | 5.234 | 0.000 | 5.659 |
| Oxycodone | 7.550 | 0.000 | 5.507 |
| 17alpha-ethynylestradiol | 4.990 | 0.000 | 5.424 |
| 1-palmitoylglycerol | 0.567 | 0.005 | 5.110 |
| Artemisinin | 4.537 | 0.000 | 5.104 |
| 4-nitroanisole | 3.072 | 0.000 | 4.790 |
| Isopropalin | 6.780 | 0.000 | 4.641 |
| Leukotriene d4 methyl ester | 4.091 | 0.022 | 4.615 |
| Zidovudine | 8.931 | 0.000 | 4.557 |
| Harmalol | 7.542 | 0.000 | 4.545 |
| Metribuzin | 6.846 | 0.000 | 4.409 |
| 6-benzylaminopurine | 2.116 | 0.046 | 4.390 |
| Vincanidine | 3.062 | 0.001 | 4.227 |
| 5-methylbenzotriazole | 0.159 | 0.014 | 4.193 |
| Olomoucine | 1.398 | 0.020 | 4.091 |
| 3'-fluoromethcathinone | 4.186 | 0.001 | 4.046 |
| Desmethylcitalopram | 4.489 | 0.001 | 3.984 |
| Fenamiphos | 8.429 | 0.000 | 3.909 |
| Metamitron | 7.673 | 0.000 | 3.671 |
| 1,5-diaminonaphthalene | 2.172 | 0.001 | 3.617 |
| All-trans-4-hydroxyretinoic acid | 4.287 | 0.000 | 3.598 |
| N.epsilon.-acetyl-l-lysine | 2.715 | 0.000 | 3.596 |
| Janerin | 5.647 | 0.000 | 3.471 |
| .gamma.-aminobutyric acid | 0.521 | 0.047 | 3.468 |
| 5-acetylamino-6-amino-3-methyluracil | 3.193 | 0.000 | 3.439 |
| Ciprofloxacin | 6.838 | 0.000 | 3.416 |
| 16.alpha.-hydroxyestrone | 6.765 | 0.015 | 3.411 |
| Diflunisal | 0.670 | 0.020 | 3.411 |
| Cinchonine | 1.546 | 0.018 | 3.239 |
| N6-me-da | 0.591 | 0.038 | 3.223 |
| Muramic acid | 4.515 | 0.000 | 3.221 |
| Perifosine | 5.828 | 0.000 | 3.173 |
| Corynoline | 8.280 | 0.002 | 3.163 |
| Iminostilbene | 2.648 | 0.001 | 3.149 |
| Bis(2-ethylhexyl) adipate | 1.954 | 0.000 | 3.143 |
| Biotin | 4.364 | 0.001 | 3.107 |
| Securinine | 3.510 | 0.002 | 3.077 |
| Nordihydroguaiaretic acid | 4.679 | 0.000 | 3.047 |
| Prostaglandin g2 | 2.364 | 0.017 | 3.041 |
| 3-hydroxymethylmefenamic acid | 4.501 | 0.000 | 3.036 |
| Shikonin | 4.600 | 0.000 | 2.991 |
| Alizarin | 0.458 | 0.025 | 2.987 |
| Pilocarpine | 5.775 | 0.000 | 2.968 |
| Norharmane | 3.029 | 0.000 | 2.945 |
| Dinotefuran | 2.793 | 0.004 | 2.941 |
| Metalaxyl | 6.410 | 0.000 | 2.926 |
| O-tolidine | 7.627 | 0.000 | 2.907 |
| N-carboxyethyl-.gamma.-aminobutyric acid | 4.604 | 0.000 | 2.838 |
| Estriol | 8.950 | 0.000 | 2.816 |
| Simvastatin 4'-methyl ether | 7.550 | 0.000 | 2.766 |
| Parthenolide | 3.486 | 0.000 | 2.735 |
| 3-cyano-7-(diethylamino)coumarin | 7.226 | 0.000 | 2.717 |
| Oxyresveratrol | 0.855 | 0.029 | 2.647 |
| 2(1h)-pyridinone | 2.460 | 0.000 | 2.633 |
| Arecoline | 5.037 | 0.000 | 2.625 |
| N,n'-diphenyl-p-phenylenediamine | 8.982 | 0.000 | 2.618 |
| Diphenylmethylphosphine | 3.950 | 0.000 | 2.586 |
| Obacunone | 0.508 | 0.009 | 2.559 |
| 4-androstene-11.beta.,17.beta.-diol-3-one | 5.995 | 0.000 | 2.535 |
| Zectran | 7.371 | 0.000 | 2.477 |
| Phe-pro | 7.081 | 0.000 | 2.440 |
| Neolinustatin | 3.307 | 0.001 | 2.432 |
| Alantolactone | 7.081 | 0.031 | 2.406 |
| Imazamox | 7.485 | 0.000 | 2.378 |
| 2,2-bis[hydroxymethyl]-2,2',2''-nitrilotriethanol | 1.550 | 0.026 | 2.378 |
| Propachlor oa | 2.761 | 0.004 | 2.376 |
| Osthole | 5.873 | 0.000 | 2.355 |
| N-(2-aminoethyl)-5-isoquinolinesulfonamide | 10.040 | 0.002 | 2.345 |
| Harmine | 4.446 | 0.000 | 2.343 |
| Promazine | 2.384 | 0.002 | 2.328 |
| Sulfamethazine | 4.044 | 0.000 | 2.324 |
| Bisphenol a diglycidyl ether | 5.260 | 0.000 | 2.289 |
| Phenanthridine | 0.602 | 0.002 | 2.272 |
| Vincamine | 3.890 | 0.000 | 2.239 |
| Brucine | 0.295 | 0.002 | 2.225 |
| Levofloxacin | 3.431 | 0.000 | 2.189 |
| (.+/-.)-.delta.9-tetrahydrocannabinol | 4.149 | 0.000 | 2.168 |
| Mescaline | 6.427 | 0.000 | 2.163 |
| (r)-.alpha.,.alpha.-diphenyl-2-pyrrolidinemethanol | 5.367 | 0.000 | 2.160 |
| Dicoumaroyl spermidine | 4.191 | 0.000 | 2.152 |
| Linolenic acid | 1.245 | 0.008 | 2.149 |
| Isoproturon | 5.703 | 0.000 | 2.148 |
| Methaqualone | 5.751 | 0.000 | 2.080 |
| Mesoporphyrin ix | 0.519 | 0.022 | 2.060 |
| Pyridaphenthion | 0.509 | 0.008 | 2.055 |
| (e)-1-[4-4-methoxyphenyl]-2-(3,5-dichlorophenyl)ethene | 9.137 | 0.000 | 2.053 |
| Reserpine | 1.654 | 0.028 | 2.020 |
| 2,5-hexanedione | 5.757 | 0.000 | 2.015 |
| 3-methyladenine | 5.421 | 0.000 | 2.004 |
| 2-arachidonoylglycerol | 6.146 | 0.000 | 1.994 |
| Nevirapine | 0.767 | 0.013 | 1.994 |
| Clozapine | 0.712 | 0.013 | 1.951 |
| Citrinin | 0.506 | 0.029 | 1.933 |
| Acetyl coenzyme a | 3.483 | 0.000 | 1.929 |
| 6.alpha./.beta.-hydroxyoxymorphone | 10.278 | 0.000 | 1.928 |
| Gelsemine | 6.036 | 0.000 | 1.926 |
| Pravadoline | 4.163 | 0.000 | 1.925 |
| Ala-Glu | 0.543 | 0.022 | 1.887 |
| N-phenylacetyl-l-prolylglycine ethyl ester | 2.083 | 0.003 | 1.885 |
| Morphine | 4.924 | 0.000 | 1.867 |
| Serotonin | 1.839 | 0.049 | 1.867 |
| Trimethoprim | 6.824 | 0.000 | 1.864 |
| Biocytin | 3.361 | 0.001 | 1.863 |
| Quinine | 2.858 | 0.001 | 1.858 |
| L-leucyl-l-proline | 3.264 | 0.017 | 1.831 |
| Cryptotanshinone | 2.950 | 0.001 | 1.812 |
| .alpha.-pyrrolidinopentiothiophenone | 4.405 | 0.005 | 1.799 |
| Piperlongumine | 7.845 | 0.000 | 1.754 |
| 6-maleimidocaproic acid | 3.111 | 0.001 | 1.743 |
| Cyclopeptine | 2.839 | 0.000 | 1.713 |
| 3,7,8,2'-tetramethoxyflavone | 0.800 | 0.047 | 1.710 |
| Fluvastatin | 6.755 | 0.000 | 1.697 |
| 1,3,4,6-tetra-o-acetyl-.beta.-d-mannopyranose | 5.214 | 0.000 | 1.693 |
| Furametpyr | 0.710 | 0.044 | 1.690 |
| Normorphine | 9.090 | 0.000 | 1.688 |
| 3-[n,n-bis(2-hydroxyethyl)amino]-2-hydroxypropanesulfonic acid | 2.330 | 0.025 | 1.677 |
| Fludrocortisone | 5.983 | 0.000 | 1.677 |
| Hexylglutathione | 0.490 | 0.000 | 1.653 |
| Ethylmorphine | 3.842 | 0.000 | 1.643 |
| Cycloxydime | 2.173 | 0.000 | 1.631 |
| 3,5-dimethoxy-4-hydroxycinnamic acid | 2.191 | 0.006 | 1.613 |
| Safranin | 0.789 | 0.036 | 1.609 |
| Ajmaline | 2.669 | 0.001 | 1.603 |
| Myclobutanil | 4.317 | 0.001 | 1.594 |
| Trans-crocetin | 1.825 | 0.014 | 1.592 |
| Lys-Cys | 3.077 | 0.000 | 1.589 |
| Pindolol | 6.409 | 0.000 | 1.587 |
| Pyridoxal | 4.445 | 0.004 | 1.586 |
| L-histidinol | 1.858 | 0.028 | 1.583 |
| Microcystin lr | 9.622 | 0.000 | 1.573 |
| Pirimicarb-desamido | 4.000 | 0.011 | 1.571 |
| Tyr-Val | 3.676 | 0.000 | 1.570 |
| Prosulfocarb | 1.796 | 0.001 | 1.564 |
| 1,3-benzenediol, 5-methyl-4-[(1r,6r)-3-methyl-6-(1-methylethenyl)-2-cyclohexen-1-yl]- | 1.424 | 0.033 | 1.557 |
| Ancymidol | 2.010 | 0.000 | 1.532 |
| 2-imino-4-methylpiperidine | 0.550 | 0.018 | 1.522 |
| Nalorphine | 5.740 | 0.000 | 1.518 |
| Tauroursodeoxycholic acid | 0.539 | 0.001 | 1.513 |
| 2-methoxy-3,5-dimethyl-6-[(4z)-4-[(e)-2-methyl-3-(4-nitrophenyl)prop-2-enylidene]oxolan-2-yl]pyran-4-one | 5.487 | 0.000 | 1.505 |
| 1-methyladenosine | 0.545 | 0.005 | 1.505 |
| N-acetyl-l-carnosine | 4.423 | 0.000 | 1.498 |
| Adrenosterone | 6.578 | 0.000 | 1.470 |
| Gibberellin a4 | 3.027 | 0.002 | 1.467 |
| Gln-val | 3.849 | 0.000 | 1.451 |
| Sepiapterin | 1.900 | 0.000 | 1.444 |
| Prednisolone | 2.322 | 0.001 | 1.443 |
| Vanillic acid diethylamide | 3.355 | 0.000 | 1.439 |
| (+)-isocorydine | 5.236 | 0.000 | 1.416 |
| Picrotin | 1.553 | 0.029 | 1.410 |
| Eremanthin | 8.104 | 0.001 | 1.408 |
| Cycloate | 2.353 | 0.005 | 1.397 |
| Val-Asp-Arg | 0.699 | 0.050 | 1.392 |
| Sethoxydim | 4.563 | 0.000 | 1.387 |
| Streptomycin a | 6.563 | 0.000 | 1.382 |
| 6-acetylmorphine | 4.557 | 0.000 | 1.370 |
| Hydroxyflutamide | 0.576 | 0.033 | 1.368 |
| Paclitaxel | 3.954 | 0.007 | 1.363 |
| Simazine | 6.903 | 0.000 | 1.357 |
| 2-hydroxyestrone | 2.171 | 0.001 | 1.355 |
| Acyclovir | 2.729 | 0.025 | 1.352 |
| N-acetylhistamine | 1.853 | 0.000 | 1.346 |
| 3-hydroxybiphenyl | 2.070 | 0.001 | 1.330 |
| Pentamidine | 0.673 | 0.001 | 1.318 |
| Cyproconazole | 1.912 | 0.001 | 1.317 |
| N-butylamine | 0.582 | 0.028 | 1.316 |
| 5-methoxyindoleacetate | 2.625 | 0.000 | 1.311 |
| Cyanazine | 1.972 | 0.014 | 1.307 |
| Leu-Trp | 2.281 | 0.001 | 1.288 |
| 4-hydroxycyclofenil | 1.802 | 0.002 | 1.274 |
| Rimsulfuron | 6.782 | 0.000 | 1.265 |
| Bisoprolol | 0.625 | 0.003 | 1.263 |
| Terbutaline | 1.828 | 0.015 | 1.249 |
| N-acetyl-d-galactosaminitol | 1.765 | 0.024 | 1.237 |
| Convolidine | 11.164 | 0.001 | 1.231 |
| 2-(4-morpholinyl)benzothiazole | 8.425 | 0.000 | 1.217 |
| 4-[[4-hydroxy-2-(2-methylpropyl)-1-oxo-3,3a-dihydro-2h-imidazo[1,2-a]indol-4-yl]methyl]-1-methyl-2,4-dihydro-1h-pyrazino[2,1-b]quinazoline-3,6-dione | 4.537 | 0.000 | 1.203 |
| Glu-Ala-Arg | 2.681 | 0.001 | 1.196 |
| Leukotriene b4 dimethylamide | 4.243 | 0.000 | 1.194 |
| 4-pregnen-6.beta.,11.beta.,17,21-tetrol-3,20-dione | 5.245 | 0.005 | 1.187 |
| Diphenylamine | 1.803 | 0.006 | 1.172 |
| Sarafloxacin | 1.774 | 0.001 | 1.165 |
| Cocaine | 7.172 | 0.000 | 1.164 |
| Bupirimate | 3.949 | 0.006 | 1.159 |
| Pyrimidifen | 5.857 | 0.002 | 1.152 |
| Vincamone | 4.749 | 0.000 | 1.150 |
| Porphobilinogen | 1.642 | 0.017 | 1.135 |
| Picoxystrobin | 0.648 | 0.019 | 1.123 |
| 1h-indazole-3-carboxylic acid, 1-(5-fluoropentyl)-, 1-naphthalenyl ester | 4.187 | 0.000 | 1.100 |
| 3-phenoxy-1-propanol | 3.151 | 0.000 | 1.099 |
| Senecionine | 10.207 | 0.000 | 1.094 |
| Dinor-12-oxophytodienoic acid | 5.172 | 0.000 | 1.079 |
| 6',7'-dihydroxybergamottin | 5.774 | 0.000 | 1.073 |
| Arginine | 1.749 | 0.002 | 1.068 |
| Prolylphenylalanine | 4.068 | 0.008 | 1.059 |
| Glu-Gln | 1.741 | 0.028 | 1.058 |
| 2-heptyl-4-hydroxyquinoline n-oxide | 3.651 | 0.007 | 1.053 |
| Malvin | 1.307 | 0.032 | 1.049 |
| .alpha.-cyclohexylmandelic acid | 2.446 | 0.000 | 1.048 |
| 13oh-terracinolide g | 3.343 | 0.042 | 1.048 |
| Coproporphyrin iii | 0.478 | 0.047 | 1.047 |
| Resmethrin | 1.659 | 0.004 | 1.039 |
| N-acetylhistidine | 1.854 | 0.002 | 1.036 |
| Dehydroaripiprazole | 1.753 | 0.047 | 1.036 |
| Lycorine | 2.880 | 0.022 | 1.031 |
| Strychnine | 3.176 | 0.000 | 1.028 |
| Epiyangambin | 8.687 | 0.000 | 1.022 |
| Chitobiose | 1.537 | 0.009 | 1.019 |
| L-bunolol | 0.688 | 0.010 | 1.016 |
| Cys-Gly-Arg | 2.391 | 0.001 | 1.014 |
| **FDG-20% Vs CON** |  |  |  |
| Sinapine | 27.054 | 0.000 | 16.819 |
| Silodosin | 12.223 | 0.000 | 13.636 |
| D-glucosaminic acid | 3.894 | 0.000 | 11.908 |
| Dethiobiotin | 15.742 | 0.000 | 11.880 |
| Citalopram | 11.812 | 0.000 | 11.502 |
| .gamma.-glu-cys | 26.383 | 0.000 | 11.400 |
| DL-tyrosine | 7.589 | 0.000 | 8.478 |
| 4,4'-diaminodiphenylmethane | 19.684 | 0.000 | 8.252 |
| Lenalidomide | 6.582 | 0.000 | 7.603 |
| Vasicinone | 12.950 | 0.000 | 7.140 |
| Olomoucine | 2.969 | 0.000 | 7.017 |
| 17alpha-ethynylestradiol | 11.249 | 0.000 | 6.629 |
| Artemisinin | 10.663 | 0.000 | 6.534 |
| 6-benzylaminopurine | 4.126 | 0.001 | 6.473 |
| Oxycodone | 17.388 | 0.000 | 6.438 |
| Isopropalin | 17.292 | 0.000 | 5.963 |
| Metribuzin | 18.462 | 0.000 | 5.648 |
| Desmethylcitalopram | 12.445 | 0.000 | 5.573 |
| 1-palmitoyl-2-linoleoyl-rac-glycerol | 0.528 | 0.044 | 5.455 |
| Benzamide, n-[2-[[(3r)-1-[trans-4-hydroxy-4-(6-methoxy-3-pyridinyl)cyclohexyl]-3-pyrrolidinyl]amino]-2-oxoethyl]-3-(trifluoromethyl)- | 1.338 | 0.023 | 5.433 |
| Vincanidine | 6.652 | 0.000 | 5.412 |
| 4-nitroanisole | 5.622 | 0.000 | 5.255 |
| Harmalol | 16.405 | 0.000 | 5.167 |
| Zidovudine | 18.573 | 0.000 | 5.086 |
| Prostaglandin g2 | 6.251 | 0.000 | 5.006 |
| 1,5-diaminonaphthalene | 4.918 | 0.000 | 5.000 |
| 3'-fluoromethcathinone | 8.767 | 0.000 | 4.818 |
| 3-oxostearic acid | 0.616 | 0.049 | 4.733 |
| Piperidine | 0.572 | 0.030 | 4.671 |
| Fenamiphos | 19.497 | 0.000 | 4.619 |
| 2-chloro-2',6'-diethylacetanilide | 0.701 | 0.026 | 4.607 |
| Benzoylecgonine | 1.615 | 0.003 | 4.601 |
| Cinchonine | 2.631 | 0.000 | 4.552 |
| Celaxanthin | 0.866 | 0.039 | 4.367 |
| Glutamic acid | 0.555 | 0.004 | 4.262 |
| Castanospermine | 0.366 | 0.001 | 4.255 |
| All-trans-4-hydroxyretinoic acid | 9.250 | 0.000 | 4.208 |
| Securinine | 8.130 | 0.000 | 4.136 |
| Ciprofloxacin | 16.150 | 0.000 | 4.051 |
| Muramic acid | 10.668 | 0.000 | 4.044 |
| Metamitron | 15.861 | 0.000 | 4.042 |
| Corynoline | 21.169 | 0.000 | 4.022 |
| Leukotriene d4 methyl ester | 4.240 | 0.000 | 4.011 |
| Dinotefuran | 6.016 | 0.000 | 3.960 |
| Janerin | 12.039 | 0.000 | 3.951 |
| 16.alpha.-hydroxyestrone | 13.137 | 0.006 | 3.941 |
| Biotin | 11.157 | 0.001 | 3.896 |
| Perifosine | 14.008 | 0.000 | 3.865 |
| Nordihydroguaiaretic acid | 10.761 | 0.000 | 3.749 |
| 4-pregnene-20.alpha.-ol-3-one | 0.620 | 0.005 | 3.748 |
| Pyrrolidine | 0.765 | 0.034 | 3.725 |
| Indirubin-3'-monoxime | 0.066 | 0.048 | 3.672 |
| 3-hydroxymethylmefenamic acid | 10.118 | 0.000 | 3.659 |
| Pilocarpine | 14.259 | 0.000 | 3.646 |
| O-tolidine | 19.779 | 0.000 | 3.602 |
| 3,3'-dimethoxybenzidine | 2.260 | 0.000 | 3.524 |
| Metalaxyl | 14.581 | 0.000 | 3.423 |
| 2,2-bis[hydroxymethyl]-2,2',2''-nitrilotriethanol | 2.858 | 0.000 | 3.390 |
| Parthenolide | 7.719 | 0.000 | 3.375 |
| Bis(2-ethylhexyl) adipate | 3.079 | 0.000 | 3.351 |
| N.epsilon.-acetyl-l-lysine | 3.773 | 0.000 | 3.334 |
| Iminostilbene | 4.315 | 0.000 | 3.298 |
| Diphenylmethylphosphine | 9.133 | 0.000 | 3.265 |
| DL-arginine | 0.506 | 0.018 | 3.257 |
| Diflunisal | 0.620 | 0.009 | 3.238 |
| Shikonin | 9.610 | 0.000 | 3.232 |
| Proscillaridin a | 1.533 | 0.013 | 3.230 |
| 2-amino-1-phenylethanol | 0.714 | 0.022 | 3.212 |
| N-alpha-acetyl-l-lysine | 0.653 | 0.012 | 3.210 |
| .gamma.-aminobutyric acid | 0.412 | 0.017 | 3.169 |
| Sulfamethazine | 10.106 | 0.000 | 3.142 |
| Estriol | 19.174 | 0.000 | 3.125 |
| Simvastatin 4'-methyl ether | 16.610 | 0.000 | 3.119 |
| Alantolactone | 15.904 | 0.004 | 3.097 |
| Harmine | 12.357 | 0.000 | 3.072 |
| D-xylose | 0.666 | 0.026 | 3.054 |
| Pyridoxine | 0.576 | 0.008 | 3.036 |
| 3-cyano-7-(diethylamino)coumarin | 14.868 | 0.000 | 2.986 |
| 2(1h)-pyridinone | 4.409 | 0.000 | 2.966 |
| Serotonin | 4.005 | 0.002 | 2.963 |
| 4-androstene-11.beta.,17.beta.-diol-3-one | 13.454 | 0.000 | 2.954 |
| N-carboxyethyl-.gamma.-aminobutyric acid | 8.159 | 0.000 | 2.918 |
| Neolinustatin | 6.530 | 0.000 | 2.916 |
| N,n'-diphenyl-p-phenylenediamine | 19.431 | 0.000 | 2.912 |
| D-glutamine | 0.559 | 0.005 | 2.895 |
| His-Leu | 3.074 | 0.001 | 2.871 |
| N-(2-aminoethyl)-5-isoquinolinesulfonamide | 25.119 | 0.000 | 2.863 |
| Osthole | 13.715 | 0.000 | 2.844 |
| Deoxyvasicinone | 2.780 | 0.000 | 2.814 |
| Arecoline | 9.532 | 0.000 | 2.799 |
| Pravadoline | 12.691 | 0.000 | 2.747 |
| 3-phenylazopyridine-2,6-diamine | 1.455 | 0.002 | 2.725 |
| 5.alpha.-pregnane-3.alpha.,20.alpha.-diol | 0.514 | 0.037 | 2.705 |
| Zectran | 14.915 | 0.000 | 2.698 |
| Norharmane | 4.371 | 0.001 | 2.676 |
| Levofloxacin | 7.431 | 0.000 | 2.648 |
| Imazamox | 15.034 | 0.000 | 2.620 |
| 3-methyladenine | 18.805 | 0.005 | 2.611 |
| Phe-pro | 13.859 | 0.000 | 2.605 |
| 3,4-dimethylmethcathinone | 1.668 | 0.042 | 2.599 |
| Succinic acid n,n-dimethylhydrazide | 0.537 | 0.038 | 2.591 |
| (e)-1-[4-4-methoxyphenyl]-2-(3,5-dichlorophenyl)ethene | 23.819 | 0.000 | 2.577 |
| (r)-.alpha.,.alpha.-diphenyl-2-pyrrolidinemethanol | 12.258 | 0.000 | 2.574 |
| 1,3-benzenediol, 5-methyl-4-[(1r,6r)-3-methyl-6-(1-methylethenyl)-2-cyclohexen-1-yl]- | 2.589 | 0.000 | 2.521 |
| Trans-crocetin | 4.266 | 0.000 | 2.518 |
| Isoproturon | 12.747 | 0.000 | 2.510 |
| Cycloxydime | 5.541 | 0.000 | 2.490 |
| Propentofylline | 3.047 | 0.000 | 2.487 |
| Biocytin | 8.047 | 0.000 | 2.472 |
| 1,3-propanediol, 2-amino-2-[2-(3-azido-4-octylphenyl)ethyl]- | 0.763 | 0.039 | 2.447 |
| Quinine | 6.281 | 0.000 | 2.446 |
| Bisphenol a diglycidyl ether | 10.183 | 0.000 | 2.444 |
| (.+/-.)-.delta.9-tetrahydrocannabinol | 8.067 | 0.000 | 2.425 |
| Gelsemine | 15.123 | 0.000 | 2.417 |
| Sulfasalazine | 0.497 | 0.010 | 2.406 |
| N-phenylacetyl-l-prolylglycine ethyl ester | 3.738 | 0.000 | 2.356 |
| Fluvastatin | 19.880 | 0.000 | 2.293 |
| Mescaline | 12.311 | 0.000 | 2.293 |
| Prosulfocarb | 4.391 | 0.000 | 2.287 |
| Acetaminophen | 0.132 | 0.031 | 2.284 |
| Propachlor oa | 4.157 | 0.000 | 2.283 |
| Isatin | 0.609 | 0.001 | 2.266 |
| 6-maleimidocaproic acid | 6.789 | 0.000 | 2.245 |
| N-acetylputrescine | 0.346 | 0.008 | 2.223 |
| Dicoumaroyl spermidine | 7.573 | 0.000 | 2.222 |
| Protriptyline | 4.236 | 0.001 | 2.221 |
| Oxyresveratrol | 1.260 | 0.006 | 2.219 |
| Cyclopeptine | 6.250 | 0.000 | 2.213 |
| N-acetyl-l-carnosine | 14.424 | 0.000 | 2.207 |
| L-leucyl-l-proline | 5.542 | 0.000 | 2.194 |
| Trans-zeatin | 1.649 | 0.004 | 2.178 |
| 2,5-hexanedione | 11.170 | 0.000 | 2.158 |
| Ne-acetyllysine | 1.732 | 0.002 | 2.126 |
| Linolenic acid | 1.491 | 0.001 | 2.090 |
| 2-methoxy-3,5-dimethyl-6-[(4z)-4-[(e)-2-methyl-3-(4-nitrophenyl)prop-2-enylidene]oxolan-2-yl]pyran-4-one | 17.830 | 0.001 | 2.088 |
| Mirtazapine n-oxide | 0.725 | 0.038 | 2.081 |
| Acephate | 0.278 | 0.046 | 2.076 |
| Morphine | 9.977 | 0.000 | 2.072 |
| Nevirapine | 0.702 | 0.007 | 2.071 |
| .alpha.-pyrrolidinopentiothiophenone | 8.944 | 0.000 | 2.059 |
| Vincamine | 5.625 | 0.000 | 2.052 |
| 6.alpha./.beta.-hydroxyoxymorphone | 20.575 | 0.000 | 2.052 |
| Trimethoprim | 12.942 | 0.000 | 2.021 |
| Myclobutanil | 9.316 | 0.000 | 1.994 |
| Piperlongumine | 16.355 | 0.000 | 1.951 |
| Picrotin | 2.766 | 0.000 | 1.949 |
| 2-arachidonoylglycerol | 10.242 | 0.000 | 1.945 |
| Tyr-Val | 8.169 | 0.000 | 1.943 |
| Cyanazine | 4.239 | 0.000 | 1.931 |
| Gibberellin a4 | 6.693 | 0.000 | 1.931 |
| Ellipticine | 3.711 | 0.000 | 1.930 |
| Ajmaline | 5.204 | 0.000 | 1.922 |
| Methaqualone | 8.363 | 0.000 | 1.922 |
| 4-hydroxycyclofenil | 4.139 | 0.000 | 1.922 |
| Labetalol | 1.758 | 0.001 | 1.893 |
| 5-acetylamino-6-amino-3-methyluracil | 3.034 | 0.048 | 1.891 |
| 2'-deoxycytidine | 1.538 | 0.028 | 1.891 |
| 3-[n,n-bis(2-hydroxyethyl)amino]-2-hydroxypropanesulfonic acid | 3.633 | 0.000 | 1.891 |
| Normorphine | 19.709 | 0.000 | 1.888 |
| Ancymidol | 3.917 | 0.000 | 1.887 |
| Gln-pro | 1.714 | 0.000 | 1.866 |
| Ponatinib | 2.086 | 0.000 | 1.865 |
| Furametpyr | 0.514 | 0.001 | 1.850 |
| Sufentanyl | 2.084 | 0.001 | 1.847 |
| Cryptotanshinone | 4.655 | 0.000 | 1.843 |
| 10-hydroxydecanoate | 9.061 | 0.006 | 1.840 |
| Ethylmorphine | 7.316 | 0.000 | 1.839 |
| Lys-Cys | 5.821 | 0.000 | 1.830 |
| Propazine | 1.527 | 0.000 | 1.827 |
| Pindolol | 14.637 | 0.000 | 1.827 |
| 1,3,4,6-tetra-o-acetyl-.beta.-d-mannopyranose | 10.005 | 0.000 | 1.819 |
| Prednisolone | 4.576 | 0.000 | 1.811 |
| 3-hydroxybiphenyl | 4.447 | 0.000 | 1.803 |
| 2-hydroxyestrone | 4.541 | 0.000 | 1.788 |
| Fenbendazole | 2.502 | 0.009 | 1.782 |
| 2-propenamide, 3-[3-[(2-benzothiazolylthio)methyl]-4-hydroxy-5-methoxyphenyl]-2-cyano- | 2.295 | 0.000 | 1.766 |
| Pantothenol | 0.418 | 0.004 | 1.755 |
| Diphenylamine | 3.876 | 0.000 | 1.749 |
| cis-9-Palmitoleic acid | 0.645 | 0.008 | 1.743 |
| 3,7,8,2'-tetramethoxyflavone | 0.768 | 0.016 | 1.736 |
| Adrenosterone | 15.106 | 0.000 | 1.736 |
| Lauryldimethylamine oxide | 0.819 | 0.003 | 1.735 |
| Granisetron | 2.676 | 0.000 | 1.719 |
| Sethoxydim | 10.819 | 0.000 | 1.718 |
| 5-methoxyindoleacetate | 5.858 | 0.000 | 1.715 |
| Nalorphine | 12.032 | 0.000 | 1.712 |
| Gln-val | 8.010 | 0.000 | 1.707 |
| Ajmalicine | 8.057 | 0.000 | 1.699 |
| Pirimicarb-desamido | 6.826 | 0.000 | 1.691 |
| Brucine | 0.312 | 0.002 | 1.681 |
| Glu-Gln | 3.900 | 0.000 | 1.670 |
| 2-imino-4-methylpiperidine | 0.268 | 0.001 | 1.665 |
| Sepiapterin | 3.304 | 0.001 | 1.661 |
| Ala-Glu | 0.549 | 0.019 | 1.654 |
| Pyrimidifen | 16.395 | 0.000 | 1.651 |
| Leu-Trp | 4.665 | 0.000 | 1.648 |
| Chitobiose | 3.372 | 0.000 | 1.643 |
| Pyridoxal | 7.755 | 0.001 | 1.621 |
| Microcystin lr | 18.611 | 0.000 | 1.615 |
| 6-acetylmorphine | 9.825 | 0.000 | 1.606 |
| Isoxadifen-ethyl | 4.609 | 0.000 | 1.595 |
| N-acetyl-d-galactosaminitol | 3.250 | 0.000 | 1.578 |
| P-toluenesulfonic acid | 0.540 | 0.009 | 1.541 |
| Rimsulfuron | 16.677 | 0.000 | 1.528 |
| Bisphenol a | 1.875 | 0.030 | 1.516 |
| Phenyl .beta.-d-galactopyranoside | 0.243 | 0.050 | 1.516 |
| Lys-Val | 0.607 | 0.039 | 1.513 |
| Leukotriene f4 | 1.510 | 0.013 | 1.510 |
| Cinobufotalin | 1.907 | 0.007 | 1.507 |
| Vincamone | 12.030 | 0.000 | 1.498 |
| Aspartame | 2.719 | 0.001 | 1.495 |
| Simetryn | 1.787 | 0.000 | 1.489 |
| Trigonelline | 5.718 | 0.002 | 1.487 |
| Simazine | 14.071 | 0.000 | 1.486 |
| Paclitaxel | 6.676 | 0.000 | 1.484 |
| 2,3-dinor-8-isoprostaglandin-f2.alpha. | 4.425 | 0.000 | 1.481 |
| Fludrocortisone | 9.009 | 0.004 | 1.469 |
| Heliotrine | 5.330 | 0.000 | 1.462 |
| 2-heptyl-4-hydroxyquinoline n-oxide | 9.121 | 0.000 | 1.461 |
| Vanillic acid diethylamide | 5.462 | 0.000 | 1.459 |
| Sarafloxacin | 3.153 | 0.000 | 1.458 |
| 4-acetamidoantipyrin | 1.967 | 0.001 | 1.449 |
| Hexylglutathione | 0.376 | 0.000 | 1.434 |
| Mestranol | 10.535 | 0.000 | 1.422 |
| N-acetylhistamine | 2.760 | 0.000 | 1.420 |
| Norfloxacin | 0.650 | 0.005 | 1.419 |
| 2-aminobenzimidazole | 0.411 | 0.037 | 1.411 |
| L-tryptophanamide | 1.391 | 0.021 | 1.399 |
| Strychnine | 8.354 | 0.000 | 1.383 |
| Estazolam | 3.220 | 0.000 | 1.382 |
| 8-hydroxy-2'-deoxyguanosine | 0.624 | 0.014 | 1.379 |
| 1h-indazole-3-carboxylic acid, 1-(5-fluoropentyl)-, 1-naphthalenyl ester | 9.887 | 0.000 | 1.368 |
| Butorphanol | 8.253 | 0.002 | 1.359 |
| Eremanthin | 11.777 | 0.000 | 1.357 |
| 4-pregnen-6.beta.,11.beta.,17,21-tetrol-3,20-dione | 11.704 | 0.001 | 1.357 |
| Aminocarb | 1.627 | 0.002 | 1.355 |
| Dinor-12-oxophytodienoic acid | 12.558 | 0.000 | 1.345 |
| Beta-estradiol | 0.784 | 0.019 | 1.336 |
| Thr-Val-Lys | 0.549 | 0.005 | 1.336 |
| Praziquantel | 0.698 | 0.024 | 1.321 |
| Mefenamic acid | 3.250 | 0.003 | 1.313 |
| Arginine | 3.052 | 0.000 | 1.313 |
| L-histidinol | 2.025 | 0.004 | 1.307 |
| Metazachlor | 1.387 | 0.016 | 1.302 |
| Flutriafol | 3.749 | 0.023 | 1.301 |
| Glu-Ala-Arg | 4.513 | 0.000 | 1.300 |
| Emetine | 1.940 | 0.001 | 1.297 |
| Pro-phe | 4.447 | 0.000 | 1.297 |
| Benz[c]acridine | 1.767 | 0.003 | 1.292 |
| Albendazole sulfone | 0.662 | 0.048 | 1.288 |
| 3,5-dimethoxy-4-hydroxycinnamic acid | 2.464 | 0.003 | 1.280 |
| Cocaine | 14.108 | 0.000 | 1.278 |
| .alpha.-cyclohexylmandelic acid | 4.839 | 0.000 | 1.275 |
| Norcodeine | 2.021 | 0.010 | 1.256 |
| Senecionine | 23.222 | 0.000 | 1.249 |
| Maltol | 0.567 | 0.049 | 1.248 |
| 2-hydroxy-3-isopropyl-6-methylbenzoic acid | 2.016 | 0.000 | 1.242 |
| 2-ethyl-2-phenylmalonamide | 2.817 | 0.048 | 1.230 |
| Leukotriene b4 dimethylamide | 7.407 | 0.000 | 1.224 |
| Lycorine | 5.976 | 0.019 | 1.223 |
| 7,8-dihydrobiopterin | 3.913 | 0.011 | 1.223 |
| Ile-Asn | 3.686 | 0.000 | 1.222 |
| 6',7'-dihydroxybergamottin | 12.523 | 0.000 | 1.221 |
| 4'-methoxychalcone | 2.261 | 0.001 | 1.220 |
| Myristicine | 6.284 | 0.000 | 1.218 |
| Curcumin | 4.700 | 0.000 | 1.213 |
| Acetyleugenol | 4.110 | 0.001 | 1.204 |
| Cortodoxone | 1.544 | 0.001 | 1.198 |
| Esculin | 0.502 | 0.025 | 1.197 |
| Triptophenolide | 7.433 | 0.001 | 1.193 |
| 2-linoleoyl-1-palmitoyl-sn-glycero-3-phosphoethanolamine | 0.518 | 0.013 | 1.192 |
| Paroxetine | 4.369 | 0.000 | 1.183 |
| 1-palmitoyl-2-oleoyl-sn-glycerol | 0.360 | 0.009 | 1.179 |
| Eurycomalactone | 5.470 | 0.000 | 1.177 |
| Tauroursodeoxycholic acid | 0.559 | 0.001 | 1.172 |
| Cyproconazole | 2.319 | 0.003 | 1.167 |
| Isoformosanine | 5.792 | 0.000 | 1.165 |
| (+)-isocorydine | 6.571 | 0.000 | 1.162 |
| Prolylphenylalanine | 7.781 | 0.002 | 1.160 |
| Streptomycin a | 8.459 | 0.000 | 1.159 |
| 5-androsten-3.beta.,16.alpha.-diol-17-one | 16.472 | 0.000 | 1.159 |
| 4-[[4-hydroxy-2-(2-methylpropyl)-1-oxo-3,3a-dihydro-2h-imidazo[1,2-a]indol-4-yl]methyl]-1-methyl-2,4-dihydro-1h-pyrazino[2,1-b]quinazoline-3,6-dione | 7.094 | 0.000 | 1.148 |
| 17-phenyltrinorprostaglandin e2 ethylamide | 13.618 | 0.000 | 1.147 |
| D-glucosamine 1-phosphate | 7.742 | 0.000 | 1.144 |
| Acyclovir | 3.022 | 0.000 | 1.142 |
| Ingenol | 1.496 | 0.004 | 1.138 |
| Convolidine | 16.278 | 0.000 | 1.134 |
| Terbutaline | 2.390 | 0.001 | 1.134 |
| Pyridoxamine | 2.784 | 0.000 | 1.132 |
| (z)-6-octadecenoic acid | 0.677 | 0.026 | 1.131 |
| Ursocholic acid | 0.901 | 0.037 | 1.130 |
| Cimicifugoside h 2 | 0.309 | 0.002 | 1.128 |
| 3-acetyl-7-diethylaminocoumarin | 3.117 | 0.000 | 1.123 |
| Bifenazate | 2.977 | 0.001 | 1.109 |
| N-acetylhistidine | 2.980 | 0.000 | 1.108 |
| Cys-Gly-Arg | 3.968 | 0.000 | 1.105 |
| Kresoxim-methyl | 1.673 | 0.001 | 1.104 |
| Benzyl butyl phthalate | 24.678 | 0.005 | 1.099 |
| Methanone, [1-(5-fluoropentyl)-1h-indol-3-yl](2,2,3,3-tetramethylcyclopropyl)- | 4.590 | 0.000 | 1.098 |
| Aminophenazone | 5.934 | 0.000 | 1.095 |
| N6-(1-iminoethyl)-l-lysine | 1.849 | 0.046 | 1.093 |
| Methyldopa | 3.713 | 0.047 | 1.089 |
| Pyrimethanil | 2.070 | 0.000 | 1.078 |
| Surfactin c | 6.069 | 0.000 | 1.071 |
| .beta.-estradiol 3-benzoate | 1.817 | 0.008 | 1.068 |
| 1-methyladenosine | 0.616 | 0.033 | 1.067 |
| 2-(4-morpholinyl)benzothiazole | 12.185 | 0.000 | 1.062 |
| Pentoxifylline | 2.170 | 0.000 | 1.061 |
| Levorphanol | 1.349 | 0.011 | 1.060 |
| Carbobenzyloxyglycylglycyl-l-norleucine | 4.812 | 0.000 | 1.049 |
| Senkirkine | 9.790 | 0.000 | 1.037 |
| Ala-Phe | 3.242 | 0.006 | 1.033 |
| 3-phenoxy-1-propanol | 4.466 | 0.000 | 1.026 |
| 2-acetyl-5-(tetrahydroxybutyl)imidazole | 4.787 | 0.000 | 1.024 |
| Pentamidine | 0.711 | 0.002 | 1.020 |
| Mepronil | 7.141 | 0.000 | 1.016 |
| Metaproterenol | 1.962 | 0.001 | 1.015 |
| Thalsimine | 0.612 | 0.001 | 1.013 |
| Rutinose | 11.482 | 0.000 | 1.005 |
| Spermidine | 1.525 | 0.003 | 1.005 |
| **FDG-20% Vs FDG-10%** |  |  |  |
| Citalopram | 0.359 | 0.005 | 10.937 |
| Dethiobiotin | 0.394 | 0.000 | 10.807 |
| D-glucosaminic acid | 0.533 | 0.000 | 9.980 |
| Silodosin | 0.604 | 0.005 | 8.666 |
| 4,4'-diaminodiphenylmethane | 0.387 | 0.000 | 7.500 |
| DL-tyrosine | 0.464 | 0.000 | 7.464 |
| Olomoucine | 0.471 | 0.000 | 7.042 |
| Benzamide, n-[2-[[(3r)-1-[trans-4-hydroxy-4-(6-methoxy-3-pyridinyl)cyclohexyl]-3-pyrrolidinyl]amino]-2-oxoethyl]-3-(trifluoromethyl)- | 0.757 | 0.025 | 6.658 |
| Vasicinone | 0.404 | 0.000 | 6.139 |
| Artemisinin | 0.425 | 0.000 | 6.007 |
| 17alpha-ethynylestradiol | 0.444 | 0.000 | 5.969 |
| 6-benzylaminopurine | 0.513 | 0.024 | 5.796 |
| Oxycodone | 0.434 | 0.000 | 5.707 |
| Isopropalin | 0.392 | 0.000 | 5.506 |
| Desmethylcitalopram | 0.361 | 0.000 | 5.401 |
| Castanospermine | 2.861 | 0.007 | 5.108 |
| Metribuzin | 0.371 | 0.004 | 5.088 |
| Lenalidomide | 0.582 | 0.013 | 5.053 |
| Prostaglandin g2 | 0.378 | 0.001 | 5.014 |
| Vincanidine | 0.460 | 0.001 | 4.957 |
| 1,5-diaminonaphthalene | 0.442 | 0.000 | 4.843 |
| Oxyresveratrol | 0.679 | 0.000 | 4.485 |
| Harmalol | 0.460 | 0.000 | 4.368 |
| Proscillaridin a | 0.580 | 0.007 | 4.359 |
| Cinchonine | 0.588 | 0.001 | 4.223 |
| 4-nitroanisole | 0.546 | 0.000 | 4.115 |
| Fenamiphos | 0.432 | 0.000 | 4.083 |
| 3'-fluoromethcathinone | 0.477 | 0.000 | 4.018 |
| Zidovudine | 0.481 | 0.001 | 3.986 |
| Securinine | 0.432 | 0.000 | 3.819 |
| Muramic acid | 0.423 | 0.000 | 3.705 |
| 3,3'-dimethoxybenzidine | 0.548 | 0.001 | 3.682 |
| Ciprofloxacin | 0.423 | 0.000 | 3.602 |
| All-trans-4-hydroxyretinoic acid | 0.463 | 0.005 | 3.506 |
| Dinotefuran | 0.464 | 0.000 | 3.493 |
| Corynoline | 0.391 | 0.002 | 3.458 |
| Biotin | 0.391 | 0.013 | 3.440 |
| Nordihydroguaiaretic acid | 0.435 | 0.000 | 3.392 |
| Metamitron | 0.484 | 0.000 | 3.361 |
| Janerin | 0.469 | 0.000 | 3.353 |
| Perifosine | 0.416 | 0.001 | 3.343 |
| Succinic acid n,n-dimethylhydrazide | 1.856 | 0.013 | 3.318 |
| O-tolidine | 0.386 | 0.000 | 3.285 |
| Pilocarpine | 0.405 | 0.000 | 3.237 |
| 3-hydroxymethylmefenamic acid | 0.445 | 0.000 | 3.208 |
| 2,2-bis[hydroxymethyl]-2,2',2''-nitrilotriethanol | 0.542 | 0.000 | 3.108 |
| 3-phenylazopyridine-2,6-diamine | 0.718 | 0.001 | 3.055 |
| Metalaxyl | 0.440 | 0.000 | 3.041 |
| Parthenolide | 0.452 | 0.000 | 3.027 |
| Propentofylline | 0.335 | 0.001 | 2.972 |
| Diphenylmethylphosphine | 0.432 | 0.000 | 2.949 |
| Celaxanthin | 0.825 | 0.047 | 2.867 |
| Harmine | 0.360 | 0.003 | 2.840 |
| Sulfamethazine | 0.400 | 0.000 | 2.783 |
| Deoxyvasicinone | 0.539 | 0.000 | 2.765 |
| Bis(2-ethylhexyl) adipate | 0.635 | 0.000 | 2.701 |
| His-Leu | 0.478 | 0.007 | 2.636 |
| Pravadoline | 0.328 | 0.000 | 2.625 |
| 4-androstene-11.beta.,17.beta.-diol-3-one | 0.446 | 0.000 | 2.621 |
| Simvastatin 4'-methyl ether | 0.455 | 0.000 | 2.610 |
| Serotonin | 0.459 | 0.016 | 2.603 |
| Prolintane | 1.443 | 0.035 | 2.588 |
| Estriol | 0.467 | 0.000 | 2.562 |
| Osthole | 0.428 | 0.000 | 2.492 |
| 3-methyladenine | 0.288 | 0.022 | 2.487 |
| 1,3-benzenediol, 5-methyl-4-[(1r,6r)-3-methyl-6-(1-methylethenyl)-2-cyclohexen-1-yl]- | 0.550 | 0.001 | 2.486 |
| Trans-zeatin | 0.646 | 0.009 | 2.473 |
| Shikonin | 0.479 | 0.005 | 2.465 |
| 2(1h)-pyridinone | 0.558 | 0.000 | 2.444 |
| Chloramphenicol succinate | 0.777 | 0.009 | 2.443 |
| Neolinustatin | 0.506 | 0.000 | 2.443 |
| Mefluidide | 1.165 | 0.018 | 2.419 |
| Cycloxydime | 0.392 | 0.000 | 2.419 |
| Iminostilbene | 0.614 | 0.001 | 2.416 |
| Trans-crocetin | 0.428 | 0.000 | 2.408 |
| 3-cyano-7-(diethylamino)coumarin | 0.486 | 0.000 | 2.391 |
| Levofloxacin | 0.462 | 0.000 | 2.377 |
| N-(2-aminoethyl)-5-isoquinolinesulfonamide | 0.400 | 0.004 | 2.375 |
| (e)-1-[4-4-methoxyphenyl]-2-(3,5-dichlorophenyl)ethene | 0.384 | 0.000 | 2.352 |
| Biocytin | 0.418 | 0.001 | 2.321 |
| N,n'-diphenyl-p-phenylenediamine | 0.462 | 0.002 | 2.276 |
| (r)-.alpha.,.alpha.-diphenyl-2-pyrrolidinemethanol | 0.438 | 0.000 | 2.274 |
| Prosulfocarb | 0.409 | 0.003 | 2.257 |
| Arecoline | 0.528 | 0.000 | 2.254 |
| 4-hydroxyquinoline | 0.432 | 0.029 | 2.248 |
| Quinine | 0.455 | 0.001 | 2.243 |
| Sufentanyl | 0.485 | 0.001 | 2.240 |
| Gelsemine | 0.399 | 0.001 | 2.217 |
| N-acetyl-l-carnosine | 0.307 | 0.000 | 2.201 |
| Gln-pro | 0.595 | 0.002 | 2.186 |
| Labetalol | 0.592 | 0.004 | 2.182 |
| Metazachlor | 0.630 | 0.000 | 2.166 |
| N-acetylputrescine | 2.062 | 0.002 | 2.149 |
| Phe-pro | 0.511 | 0.000 | 2.125 |
| Fluvastatin | 0.340 | 0.000 | 2.122 |
| Propazine | 0.669 | 0.000 | 2.112 |
| Isoproturon | 0.447 | 0.001 | 2.079 |
| N-phenylacetyl-l-prolylglycine ethyl ester | 0.557 | 0.001 | 2.063 |
| Amphetamine | 2.458 | 0.045 | 2.048 |
| Imazamox | 0.498 | 0.003 | 2.044 |
| N-carboxyethyl-.gamma.-aminobutyric acid | 0.564 | 0.015 | 2.043 |
| Cyclopeptine | 0.454 | 0.000 | 2.041 |
| 2-methoxy-3,5-dimethyl-6-[(4z)-4-[(e)-2-methyl-3-(4-nitrophenyl)prop-2-enylidene]oxolan-2-yl]pyran-4-one | 0.308 | 0.007 | 2.036 |
| 6-maleimidocaproic acid | 0.458 | 0.000 | 2.035 |
| (.+/-.)-.delta.9-tetrahydrocannabinol | 0.514 | 0.000 | 1.987 |
| L-tryptophanamide | 0.665 | 0.012 | 1.970 |
| 2-propenamide, 3-[3-[(2-benzothiazolylthio)methyl]-4-hydroxy-5-methoxyphenyl]-2-cyano- | 0.484 | 0.000 | 1.970 |
| Ellipticine | 0.440 | 0.000 | 1.943 |
| Lauryldimethylamine oxide | 1.206 | 0.006 | 1.933 |
| Zectran | 0.494 | 0.010 | 1.915 |
| Bisphenol a diglycidyl ether | 0.517 | 0.001 | 1.884 |
| 4-hydroxycyclofenil | 0.435 | 0.000 | 1.877 |
| Indirubin-3'-monoxime | 3.544 | 0.024 | 1.870 |
| Cyanazine | 0.465 | 0.000 | 1.845 |
| Pantothenol | 2.169 | 0.043 | 1.824 |
| Granisetron | 0.465 | 0.000 | 1.822 |
| Cyclocurcumin | 0.490 | 0.013 | 1.784 |
| Mescaline | 0.522 | 0.000 | 1.771 |
| Gibberellin a4 | 0.452 | 0.001 | 1.768 |
| Tyr-Val | 0.450 | 0.000 | 1.759 |
| Ponatinib | 0.620 | 0.001 | 1.756 |
| Ajmalicine | 0.318 | 0.001 | 1.756 |
| Morphine | 0.494 | 0.000 | 1.743 |
| Promazine | 1.567 | 0.015 | 1.738 |
| 2,5-hexanedione | 0.515 | 0.000 | 1.724 |
| Ingenol | 0.573 | 0.001 | 1.724 |
| Diphenylamine | 0.465 | 0.000 | 1.716 |
| Val-Asp-Arg | 0.523 | 0.008 | 1.697 |
| Ancymidol | 0.513 | 0.000 | 1.684 |
| 3-hydroxybiphenyl | 0.466 | 0.000 | 1.680 |
| Ajmaline | 0.513 | 0.001 | 1.672 |
| Chitobiose | 0.456 | 0.000 | 1.671 |
| Phenanthridine | 0.629 | 0.002 | 1.648 |
| Picrotin | 0.562 | 0.001 | 1.635 |
| 2-hydroxyestrone | 0.478 | 0.000 | 1.634 |
| Acetyl coenzyme a | 2.172 | 0.001 | 1.634 |
| Prednisolone | 0.507 | 0.000 | 1.633 |
| 5-methoxyindoleacetate | 0.448 | 0.000 | 1.609 |
| Isoxadifen-ethyl | 0.414 | 0.000 | 1.599 |
| Simetryn | 0.621 | 0.002 | 1.598 |
| Bergamotin | 0.622 | 0.002 | 1.594 |
| Dicoumaroyl spermidine | 0.553 | 0.003 | 1.586 |
| Pyrimidifen | 0.357 | 0.000 | 1.584 |
| Normorphine | 0.461 | 0.000 | 1.579 |
| Glu-Gln | 0.446 | 0.000 | 1.554 |
| Piperlongumine | 0.480 | 0.001 | 1.547 |
| Myclobutanil | 0.463 | 0.007 | 1.546 |
| Norcodeine | 0.495 | 0.012 | 1.542 |
| Adrenosterone | 0.435 | 0.000 | 1.538 |
| Glu-Asp-Arg | 0.535 | 0.010 | 1.536 |
| Trimethoprim | 0.527 | 0.000 | 1.533 |
| Leu-Trp | 0.489 | 0.000 | 1.527 |
| N-.alpha.-(tert-butoxycarbonyl)-l-histidine | 0.696 | 0.011 | 1.524 |
| Pindolol | 0.438 | 0.001 | 1.523 |
| Benz[c]acridine | 0.590 | 0.006 | 1.522 |
| Sethoxydim | 0.422 | 0.000 | 1.521 |
| Lopinavir | 0.581 | 0.001 | 1.509 |
| N.epsilon.-acetyl-l-lysine | 0.720 | 0.013 | 1.502 |
| 1,3,4,6-tetra-o-acetyl-.beta.-d-mannopyranose | 0.521 | 0.000 | 1.498 |
| 2,3-dinor-8-isoprostaglandin-f2.alpha. | 0.417 | 0.000 | 1.496 |
| .beta.-estradiol 3-benzoate | 0.457 | 0.003 | 1.489 |
| 6.alpha./.beta.-hydroxyoxymorphone | 0.500 | 0.006 | 1.463 |
| Ethylmorphine | 0.525 | 0.000 | 1.459 |
| Kresoxim-methyl | 0.559 | 0.000 | 1.443 |
| Norfloxacin | 1.322 | 0.016 | 1.441 |
| .alpha.-pyrrolidinopentiothiophenone | 0.493 | 0.018 | 1.428 |
| L-saccharopine | 0.765 | 0.031 | 1.428 |
| Gln-val | 0.481 | 0.000 | 1.421 |
| L-leucyl-l-proline | 0.589 | 0.041 | 1.418 |
| Nalorphine | 0.477 | 0.000 | 1.409 |
| Mestranol | 0.303 | 0.000 | 1.407 |
| Estazolam | 0.451 | 0.001 | 1.407 |
| Trigonelline | 0.400 | 0.014 | 1.398 |
| Butorphanol | 0.296 | 0.014 | 1.391 |
| Sepiapterin | 0.575 | 0.018 | 1.385 |
| Heliotrine | 0.423 | 0.000 | 1.382 |
| Lys-Cys | 0.529 | 0.001 | 1.363 |
| 6-acetylmorphine | 0.464 | 0.000 | 1.356 |
| N-acetyl-d-galactosaminitol | 0.543 | 0.006 | 1.354 |
| Thr-Val-Lys | 1.553 | 0.044 | 1.352 |
| 2-arachidonoylglycerol | 0.600 | 0.000 | 1.347 |
| 2-heptyl-4-hydroxyquinoline n-oxide | 0.400 | 0.001 | 1.337 |
| Levorphanol | 0.760 | 0.018 | 1.334 |
| Vincamone | 0.395 | 0.003 | 1.332 |
| Strychnine | 0.380 | 0.006 | 1.324 |
| Sarafloxacin | 0.563 | 0.004 | 1.322 |
| Prometryne | 0.803 | 0.014 | 1.316 |
| Rimsulfuron | 0.407 | 0.003 | 1.314 |
| 2-linoleoyl-1-palmitoyl-sn-glycero-3-phosphoethanolamine | 1.697 | 0.006 | 1.313 |
| 7,8-dihydrobiopterin | 0.347 | 0.023 | 1.312 |
| Aspartame | 0.537 | 0.007 | 1.308 |
| N1-(3-aminopropyl)-n1-methylpropane-1,3-diamine | 0.430 | 0.043 | 1.294 |
| 2-imino-4-methylpiperidine | 2.052 | 0.001 | 1.291 |
| Pyrimethanil | 0.505 | 0.001 | 1.282 |
| Paroxetine | 0.377 | 0.001 | 1.274 |
| Perfluorooctyl phosphate | 0.811 | 0.044 | 1.248 |
| 1h-indazole-3-carboxylic acid, 1-(5-fluoropentyl)-, 1-naphthalenyl ester | 0.423 | 0.005 | 1.219 |
| Linolenic acid | 0.835 | 0.029 | 1.214 |
| Simazine | 0.491 | 0.000 | 1.209 |
| Mefenamic acid | 0.504 | 0.020 | 1.207 |
| 2-hydroxy-3-isopropyl-6-methylbenzoic acid | 0.643 | 0.000 | 1.198 |
| 3-[n,n-bis(2-hydroxyethyl)amino]-2-hydroxypropanesulfonic acid | 0.641 | 0.047 | 1.196 |
| 17-phenyltrinorprostaglandin e2 ethylamide | 0.282 | 0.001 | 1.180 |
| Vincamine | 0.692 | 0.037 | 1.174 |
| Curcumin | 0.425 | 0.005 | 1.174 |
| Cortodoxone | 0.739 | 0.010 | 1.174 |
| Pro-phe | 0.491 | 0.002 | 1.174 |
| Dinor-12-oxophytodienoic acid | 0.412 | 0.000 | 1.157 |
| Cryptotanshinone | 0.634 | 0.022 | 1.139 |
| Myristicine | 0.434 | 0.000 | 1.128 |
| Microcystin lr | 0.517 | 0.002 | 1.121 |
| Isobutyric acid | 1.145 | 0.024 | 1.112 |
| Met-Met-Arg | 0.130 | 0.000 | 1.103 |
| Bifenazate | 0.502 | 0.005 | 1.098 |
| .alpha.-cyclohexylmandelic acid | 0.505 | 0.000 | 1.097 |
| Surfactin c | 0.381 | 0.000 | 1.088 |
| Senecionine | 0.440 | 0.000 | 1.078 |
| 4'-methoxychalcone | 0.593 | 0.008 | 1.076 |
| 4,5-epoxy-7z,10z,13z,16z,19z-docosapentaenoic acid, methyl ester | 1.263 | 0.022 | 1.076 |
| Arginine | 0.573 | 0.001 | 1.073 |
| Secbumeton | 0.568 | 0.032 | 1.066 |
| 6',7'-dihydroxybergamottin | 0.461 | 0.000 | 1.061 |
| Phenanthroline | 0.671 | 0.003 | 1.060 |
| N-acetylhistamine | 0.672 | 0.001 | 1.058 |
| Ile-Asn | 0.553 | 0.000 | 1.057 |
| Vanillic acid diethylamide | 0.614 | 0.002 | 1.051 |
| Pirimicarb-desamido | 0.586 | 0.046 | 1.049 |
| Methaqualone | 0.688 | 0.004 | 1.035 |
| Aminophenazone | 0.444 | 0.000 | 1.027 |
| 5-androsten-3.beta.,16.alpha.-diol-17-one | 0.432 | 0.000 | 1.022 |
| Isoformosanine | 0.494 | 0.000 | 1.021 |
| Methanone, [1-(5-fluoropentyl)-1h-indol-3-yl](2,2,3,3-tetramethylcyclopropyl)- | 0.484 | 0.000 | 1.017 |
| Codeine | 0.493 | 0.005 | 1.013 |
| Cocaine | 0.508 | 0.000 | 1.009 |
| 3-amino-2,3-dihydrobenzoic acid | 0.693 | 0.010 | 1.002 |
| 1-palmitoyl-2-oleoyl-sn-glycerol | 2.089 | 0.025 | 1.001 |

Note: VIP, variable influence on projection; FC, fold change; CON, FDG-10% and FDG-20% for the basal diet group, the FDG replacing 10% concentrate group, and the FDG replacing 20% concentrate group, respectively.


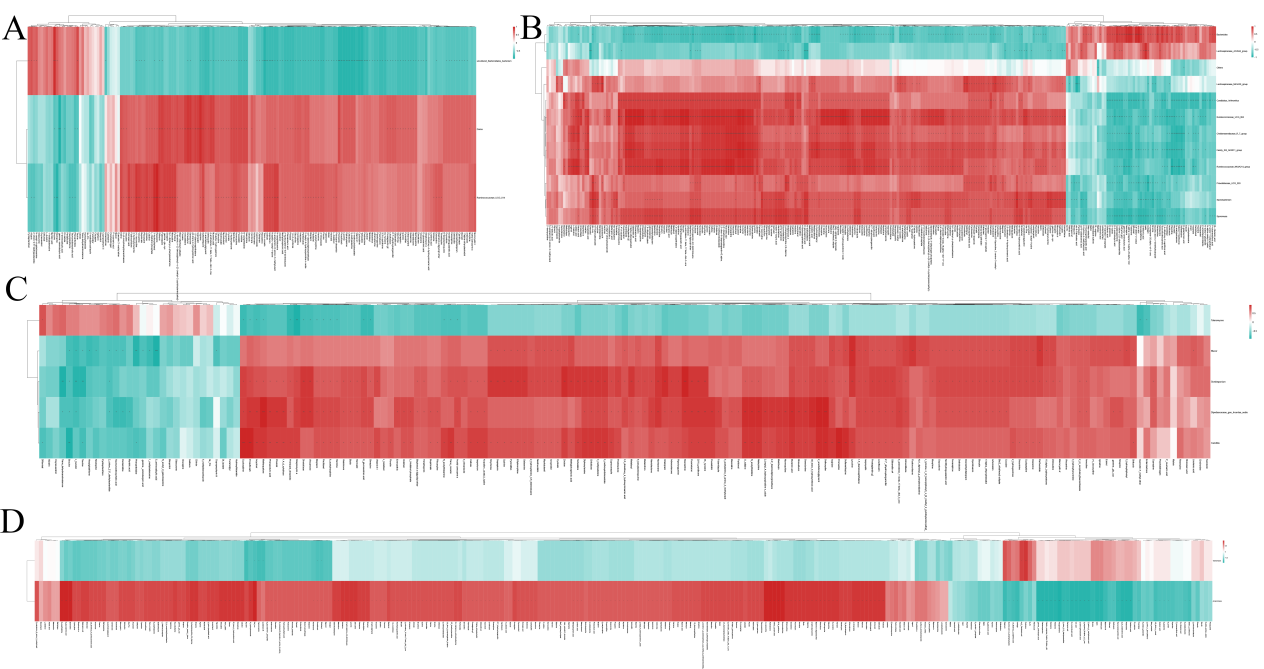


Fig. S1 Correlation analysis. (A-B)Correlation analysis between significantly different intestinal bacterial communities and significantly different metabolites in the FDG-10% vs CON group and FDG-20% vs CON group. (C-D)Correlation analysis between significantly different intestinal fungal communities and significantly different metabolites in the FDG-10% vs CON and FDG-20% vs CON groups. Red and blue colors indicate positive and negative correlations, respectively, and the color scale indicates the magnitude of the correlation coefficient. Significant correlations are *, 0.01 < *P* ≤ 0.05, **, 0.001 < *P* ≤ 0.01 and ***, *P* < 0.001.
